# Brain cone-beam CT image quality improvement using a deep-learning-based denoising method: a multicenter retrospective study

**DOI:** 10.1007/s00234-026-04148-9

**Published:** 2026-08-24

**Authors:** Fredrik Ståhl, Nicole M. Cancelliere, Jens Kolloch, Shobhit Mathur, Ibrahim Abdulaziz Almulhim, Kevin Janot, Vamsi Gontu, Nikolas D. Schnellbächer, Marijke van Vlimmeren, Erik Hummel, Vitor M. Pereira, Anna Falk Delgado

**Affiliations:** 1 Department of Clinical Neuroscience, Karolinska Institutet, Stockholm, Sweden; 2 RADIS Lab, Li Ka Shing Knowledge Institute, Unity Health Toronto, Toronto, Ontario Canada; 3 Department of Neuroradiology, Karolinska University Hospital, Stockholm, Sweden; 4 Departments of Neurosurgery and Medical Imaging, St. Michael’s Hospital, Toronto, Canada; 5 Department of Neuroradiology, Centre Hospitalier Universitaire de Tours, Tours, France; 6 Philips (Germany), Hamburg, Germany; 7 Philips (Netherlands), Best, Netherlands

**Keywords:** Brain, Stroke, Deep learning, Cone-beam CT

## Abstract

**Purpose:**

Deep learning (DL) denoising may improve cone-beam CT (CBCT) image quality for point-of-care stroke assessment in the interventional suite. The purpose of this study was to evaluate the impact of a DL-based denoising algorithm on objective and subjective image quality in brain CBCT using both standard circular and advanced dual-axis trajectories.

**Methods:**

We retrospectively analyzed 20 noncontrast brain CBCT acquisitions (Karolinska: 10 standard circular; St Michael's: 10 dual-axis). A DL-based denoising algorithm was applied at three strengths (Minimal, Medium, High) and compared to standard images with no additional denoising. Objective metrics (noise, signal-to-noise ratio (SNR), contrast-to-noise ratio (CNR), artifact indices) were measured using standardized ROIs. Six experts rated subjective image quality on 5-point Likert scales. Paired tests with Bonferroni correction were used for comparisons.

**Results:**

High-level DL denoising significantly improved all objective metrics (noise, SNR, CNR, artifact indices) for both thin and thick slices (all *p*<.001). It doubled gray–white matter CNR (thin slices: 2.31 vs. 1.08), reduced noise, and improved subcalvarial and posterior fossa artifact indices. Subjectively, high-level denoising yielded higher median ratings for noise, texture, sharpness, brain parenchyma visualization, CSF spaces, and confidence in assessing ischemia and hemorrhage (all *p*<.001). Improvements were consistent for both acquisition techniques, and perceived artifact severity did not differ (*p*>.99). Inter-reader agreement was substantial.

**Conclusion:**

The DL-based denoising algorithm significantly improved objective and subjective brain CBCT image-quality; no difference in perceived artifact severity was detected. These findings support further evaluation of deep learning-enhanced CBCT denoising for brain imaging in the interventional suite.

**Supplementary Information:**

The online version contains supplementary material available at https://doi.org/10.1007/s00234-026-04148-9.

## Introduction

Cone-Beam CT (CBCT) is extensively utilized in interventional neuroradiology for CT-like images of the brain [1]. CBCT can serve as an alternative to conventional non-contrast CT to expedite stroke treatment, but its historically inferior image quality has limited its utility [2–4]. Recent advancements suggest the latest generation of CBCT can enable reliable stroke assessment directly in the interventional suite [5–7]. Altering the CBCT image acquisition by scanning the brain with a non-circular beam trajectory further improves diagnostic confidence by reducing cone-beam and bone artifacts [8, 9]. As image acquisition typically takes several seconds, the addition of advanced post-processing to correct possible motion artifacts further increases the robustness of CBCT imaging [10].

Iterative model reconstruction is known to reduce noise and improve contrast-to-noise ratio in brain CT, compared to traditional filtered back projection [11]. Image reconstruction utilizing deep-learning-based (DL) approaches has gained interest in recent years, showing inherent benefits to image quality in non-contrast brain CT as well as other parts of the body, compared to iterative reconstruction approaches [12–14]. Employing DL-based denoising in CBCT, which typically utilizes Feldkamp-Davis-Kress reconstruction [15], could render images of improved quality and diagnostic confidence. The deep-learning-based algorithm used in this context is based on a deep neural network algorithm, which is trained in a supervised manner to reproduce the noise of routine-dose acquisitions, when compared to simulated low-dose reconstructions [16]. In this study, we evaluate if a standard brain CBCT reconstruction benefits from an added DL-denoising algorithm in terms of objective and subjective image quality, both using a standard circular image acquisition technique as well as a state-of-the-art non-circular acquisition technique with motion artifact correction.

## Materials and methods

### Participants

This retrospective two-center image-quality study included 20 anonymized non-contrast brain CBCT examinations. Ten examinations were selected by random sampling from each eligible site dataset (Karolinska University Hospital, *n* = 29; St Michael's Hospital, *n* = 56). Each examination generated eight image sets: four processing conditions (standard reconstruction, Minimal-, Medium-, and High-level DL-based denoising) in two slice formats. The Karolinska images were acquired after obtaining written informed consent and approved by the Swedish Ethical Review Authority (approval number: 2020-04467). Anonymizing the image material for use in the current study was approved by the same authority with approval number: 2023-06021-02. The Karolinska dataset consisted of ten randomized sets of non-contrast brain scans from the original study, which prospectively included consecutive stroke patients aged 50 or older [7]. The images from St Michael's Hospital were acquired after obtaining research ethics board approval from the Unity Health Research Ethics Board (REB #26-017) and included the use of images retrospectively collected from stroke patients aged 50 or older undergoing neurointerventional procedures. No formal sample size calculation was performed for this retrospective multicenter study.

### Imaging protocol & post processing

Using Allura Xper FD20/15 angiography systems (Philips Healthcare), all standard circular acquisitions were obtained at Karolinska University Hospital (Stockholm, Sweden), whereas all dual-axis acquisitions with motion-artifact correction were obtained at St Michael's Hospital (Toronto, Canada) [8]. The baseline reconstructions (no DL-based denoising applied) were state-of-the art SmartCT SoftTissue cone-beam CT reconstructions (SmartCT, Philips Healthcare). Radiation doses were comparable (see scan details in Supplemental Table 1).

As an additional post-processing step, a DL-based denoising algorithm was applied in the image domain for all scans. Denoising was applied to the images using three different strengths (Minimal, Medium, and High), in comparison to default reconstructions without DL-based denoising applied (see details on denoising strength in the Supplementary Information). The DL-based denoising solution used for this study is a vendor-supplied algorithm (Philips Healthcare). Neural network training was carried out in a supervised manner, comparing normal dose routine reconstructions against simulated low-dose scans, producing exactly matching training pairs. Noise simulation was dominantly accounting for the quantum noise of a low-dose X-ray acquisition and was performed similar to [17]. The network was configured to predict the residual noise in each training sample (residual learning). Once trained, the final denoising level/strength can be controlled by a residual blending mechanism. Additional information regarding the DL algorithm is provided in the Supplementary Information. None of the CBCT image data included in this study was used for model training or any other form of model fine-tuning.

### Objective image analysis

Objective image quality was evaluated using circular ROIs on axial slices, consistent with prior brain CT and CBCT studies [7, 18, 19]. Noise, SNR, and CNR for gray and white matter were obtained using 25 mm² ROIs in cortical and juxtacortical white matter regions at the level of the basal ganglia, and in the thalamus and posterior limb of the internal capsule. To evaluate image artifacts, a 25 mm² ROI was positioned near the skull bone at the level of the basal ganglia (“Subcalvarial”), and a 200 mm² ROI in the interpetrous area of the posterior fossa. See details on the calculations of noise, SNR, CNR, and artifact indices in the Supplementary Information. For the artifact indices, a lower value indicated a lower degree of image artifacts (such as beam hardening) in the specified region.

### Subjective image analysis

Images were presented as thin contiguous slices (0.66 mm) or normal diagnostic image slabs (5 mm thick contiguous slices, average intensity projection) in axial and sagittal orientation in a 1 × 2 layout. Six readers, equally divided between Karolinska University Hospital and St Michael's Hospital (3 interventional radiologists and 3 diagnostic radiologists (F.S., J.K., S.M., I.A.A., K.J., and V.G.), with a minimum of 7 years of clinical experience in stroke imaging), individually assessed 160 anonymized image sets in a computer-generated randomized and blinded single sequence (20 scans, 4 noise levels, 2 slice thicknesses) without any clinical information. No washout interval was imposed between alternative versions of the same examination. Window width and level were preset to 100/25 but could be modified at the reader’s discretion.

Using Likert scales ranging from 1 to 5, the readers were asked to rate the overall impression of the noise level (high to low), image texture (undesired to excellent), and image sharpness (blurred to excellent). They were also asked to rate their impression of the brain parenchyma in five locations, the delineation of spaces containing cerebrospinal fluid (CSF) in four locations, perceived artifact severity and impact, and confidence in assessing anterior circulation ischemia or intraparenchymal hemorrhage. A score of 5 was the most favorable, and 1 was the least favorable in all categories. The full questionnaire and anchors are provided in the Supplementary Information.

### Statistical analysis

Continuous objective metrics were summarized as mean (SD) and compared between paired processing conditions using two-sided paired t tests. Ordinal reader ratings and paired rating differences were summarized as median (IQR) and compared using two-sided Wilcoxon matched-pairs signed-rank tests. For each outcome and slice format, prespecified pairwise contrasts compared the High denoising setting with each of the other reconstruction conditions: standard reconstruction without additional denoising, Minimal denoising, and Medium denoising. Bonferroni correction was applied across the three contrasts within each outcome and slice format. Reported *p* values are Bonferroni-adjusted, and adjusted *p* < .05 was considered statistically significant. There were no missing objective measurements or reader ratings, and no imputation was performed.

Interreader agreement among all six readers was assessed separately for thin- and thick-slice formats using multi-rater Gwet AC2 with ordinal weights applied to the original 1–5 ratings across all four reconstruction conditions [20]. Center-specific analyses were exploratory and descriptive; no center-by-denoising interaction test was performed. For outcomes assessed at multiple anatomic locations, the original location-level ratings were pooled. 95% confidence intervals were obtained from 20,000 bias-corrected and accelerated bootstrap resamples clustered by examination. Statistical analyses were conducted in RStudio (v2024.09.1 + 394 Posit Software PBC, Boston, MA, USA, and R v4.4.2, The R Foundation for Statistical Computing).

## Results

**Fig. 1:**
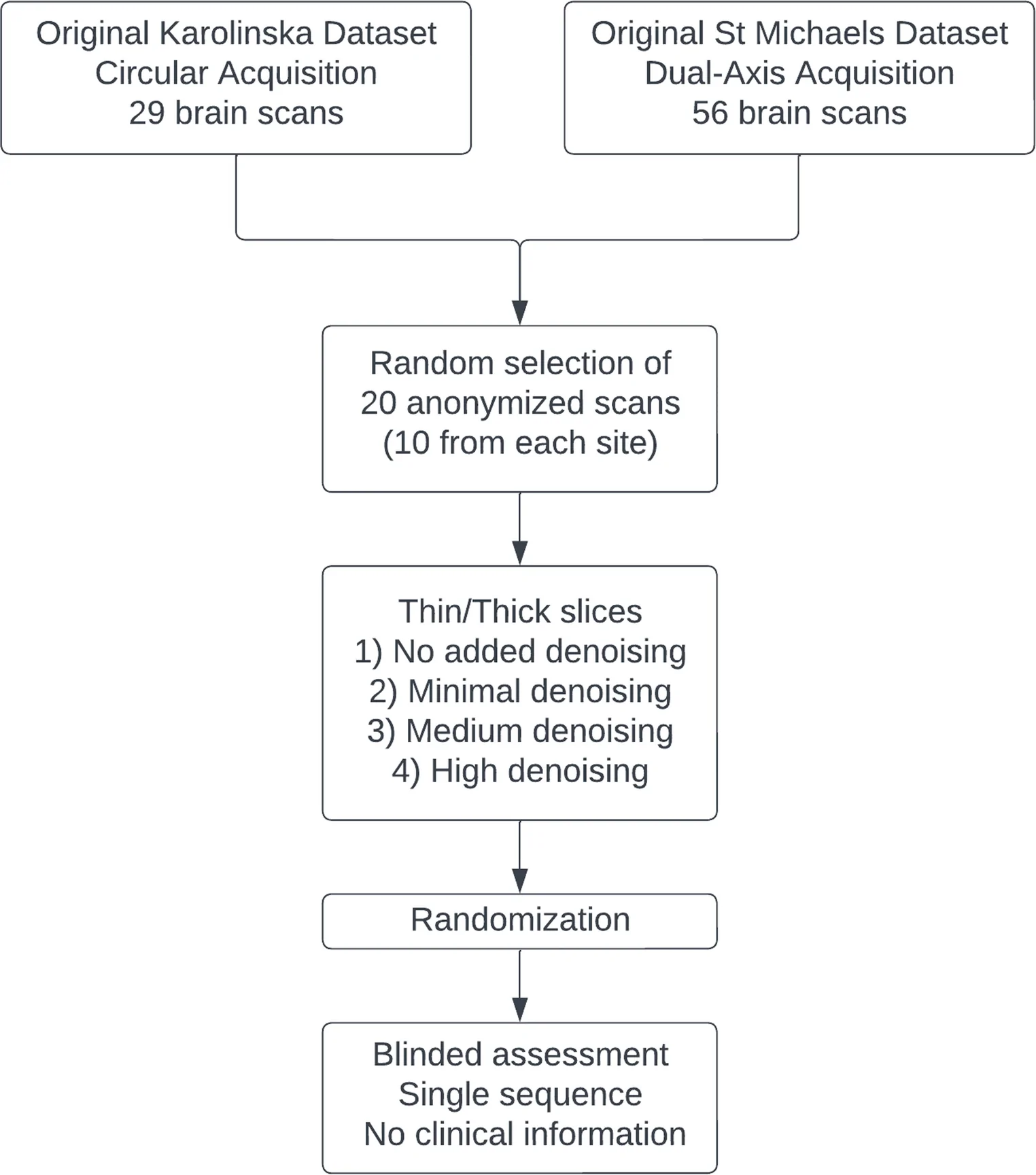
Flow diagram showing random selection of 10 examinations from each site, generation of eight image sets per examination, computer-randomized presentation order, and blinded independent assessment

At Karolinska University Hospital, mean age was 74 years (SD 9) and 6/10 participants (60%) were female; at St Michael's Hospital, mean age was 74 years (SD 13) and 8/10 (80%) were female. Examinations were acquired immediately before or within 24 h after neurovascular intervention. Table 1 summarizes demographic and clinical characteristics; Fig. 1 shows examination selection, image-set generation, randomization, and blinded review. All examinations were included in the analyses; none was excluded after sampling.

**Table 1:** Key demographic and clinical characteristics

Characteristic	Karolinska images *(n = 10)*	St Michael's images *(n = 10)*
Age, years (mean ± SD)	74 ± 9	74 ± 13
Female	6 (60%)	8 (80%)
Right hemisphere affected	6 (60%)	6 (60%)
ICA occlusion	2 (20%)	3 (30%)
MCA occlusion	8 (80%)	6 (60%)
AcomA aneurysm	0 (0%)	1 (10%)
ASPECTS (IQR)	8.5 (7–10)	8.5 (8–10)

Note: Age is presented as mean ± SD; ASPECTS is presented as median (IQR). ASPECTS = Alberta Stroke Program Early CT Score; ICA = internal carotid artery; MCA = middle cerebral artery; AcomA = anterior communicating artery

### Objective image quality

**Fig. 2:**
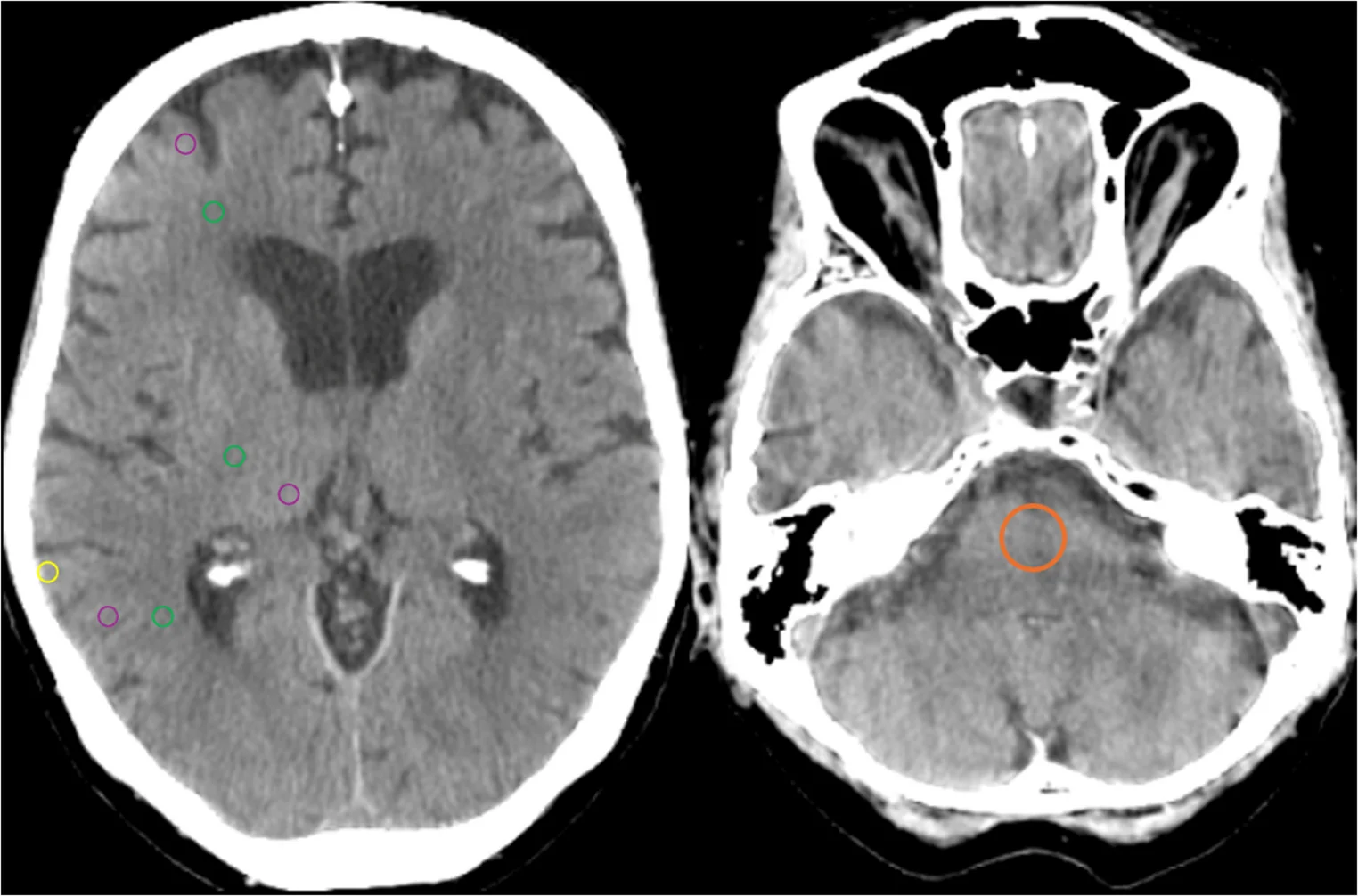
Brain cone-beam computed tomography image showing representative ROI placement in the clinically unaffected hemisphere and posterior fossa: gray matter (magenta), adjacent white matter (green), subcalvarial region (yellow), and posterior fossa (orange). This illustrative High-level denoised 0.66-mm image is displayed at window width/level 100/25

**Fig. 3:**
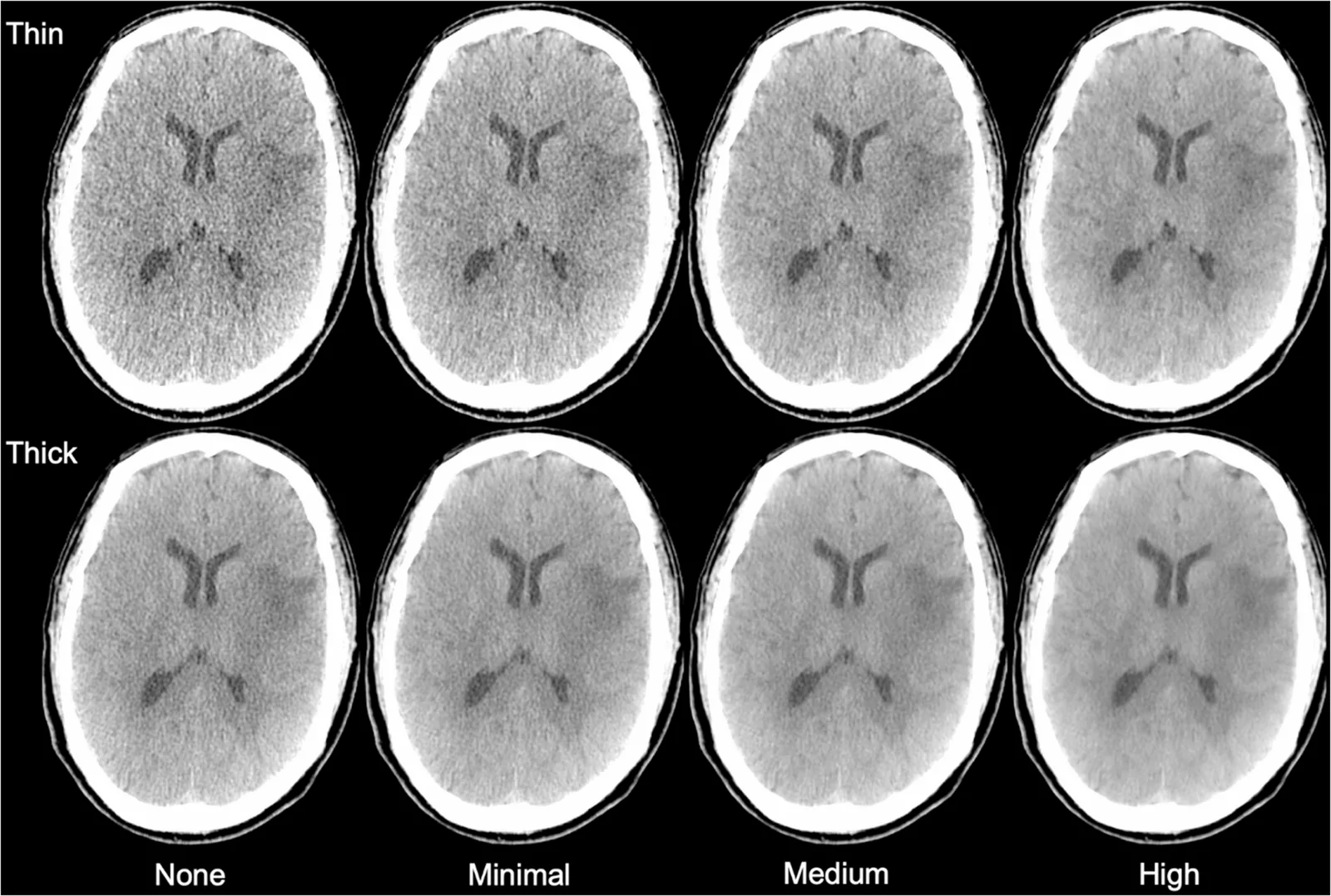
Representative CBCT image examples of a patient with a left-hemispheric ischemic stroke, shown with thin slices (0.66 mm, top row) and thick slices (5 mm, bottom row). Increasing levels of denoising are applied from left to right, ranging from No additional denoising (far left) to High-level denoising (far right). Window/Level 100/25

Objective metrics changed monotonically with increasing denoising strength (Supplemental Table 2). Compared with the native standard reconstruction, High-level denoising improved every objective metric in both slice formats (all adjusted *p*<.001; Table 2). In thin slices, gray-white matter CNR increased from 1.08 (SD 0.20) to 2.31 (SD 0.39), the subcalvarial artifact index decreased from 9.46 (SD 2.11) to 6.73 (SD 2.05), and the posterior-fossa artifact index decreased from 12.41 (SD 2.43) to 7.21 (SD 2.03). Comparisons of High denoising versus standard reconstruction, Minimal- and Medium denoising were significant for every objective metric and slice format (all Bonferroni-adjusted *p*<.001; Supplemental Table 2). Figures 2 and 3 show ROI placement and representative images.

**Table 2:** Objective Image Quality

Metric and slice thickness	High denoising [mean (SD)]	None added (standard) [mean (SD)]	*P*-value (adj)
Gray matter noise			
Thick (5 mm)	2.33 (0.39)	4.54 (0.70)	*p<.001*
Thin (0.66 mm)	4.04 (0.61)	8.48 (1.34)	*p<.001*
White matter noise			
Thick (5 mm)	2.15 (0.41)	4.55 (0.81)	*p<.001*
Thin (0.66 mm)	3.78 (0.62)	8.52 (1.48)	*p<.001*
Gray matter SNR			
Thick (5 mm)	15.68 (4.73)	7.94 (2.29)	*p<.001*
Thin (0.66 mm)	9.10 (2.77)	4.32 (1.23)	*p<.001*
White matter SNR			
Thick (5 mm)	13.63 (4.84)	6.11 (2.07)	*p<.001*
Thin (0.66 mm)	7.58 (2.91)	3.25 (1.22)	*p<.001*
Gray vs. white matter CNR			
Thick (5 mm)	3.86 (0.84)	1.91 (0.42)	*p<.001*
Thin (0.66 mm)	2.31 (0.39)	1.08 (0.20)	*p<.001*
Subcalvarial artifact index			
Thick (5 mm)	5.88 (2.03)	6.95 (2.05)	*p<.001*
Thin (0.66 mm)	6.73 (2.05)	9.46 (2.11)	*p<.001*
Posterior fossa artifact index			
Thick (5 mm)	4.77 (1.63)	7.31 (1.71)	*p<.001*
Thin (0.66 mm)	7.21 (2.03)	12.41 (2.43)	*p<.001*

Note: “Subcalvarial” denotes a region adjacent to the skull at the basal ganglia level. *P* values compare High-level denoising with the native standard reconstruction and remain significant after Bonferroni correction across the three prespecified High-level contrasts per outcome and slice format. Noise and artifact indices are in Hounsfield units (HU); SNR and CNR are unitless

### Subjective image quality

**Fig. 4:**
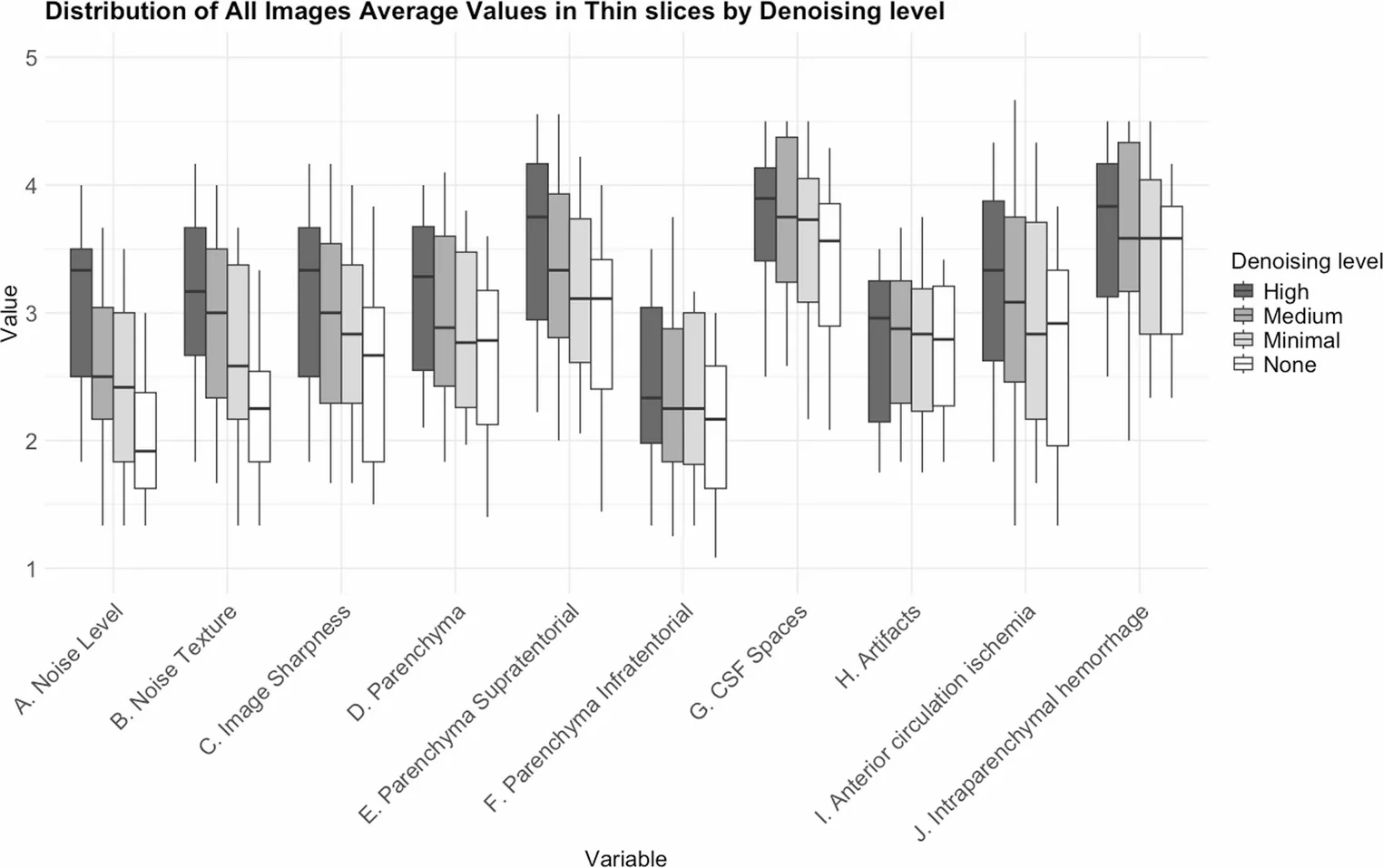
Boxplots showing the distribution of thin-slice Likert ratings across readers and examinations for each processing condition. Center lines indicate medians, boxes interquartile ranges, and whiskers extend to 1.5 times the interquartile range. “None” denotes the standard reconstruction without added deep-learning denoising. Exact Bonferroni-adjusted *p* values for the three prespecified High-level contrasts are reported in Supplemental Table 3

For thin slices, High-level denoising had the highest median rating in every category (Fig. 4). Median ratings were generally 3–4, whereas infratentorial parenchyma was rated 2–3. Median perceived artifact-severity ratings were similar across processing conditions.

**Fig. 5:**
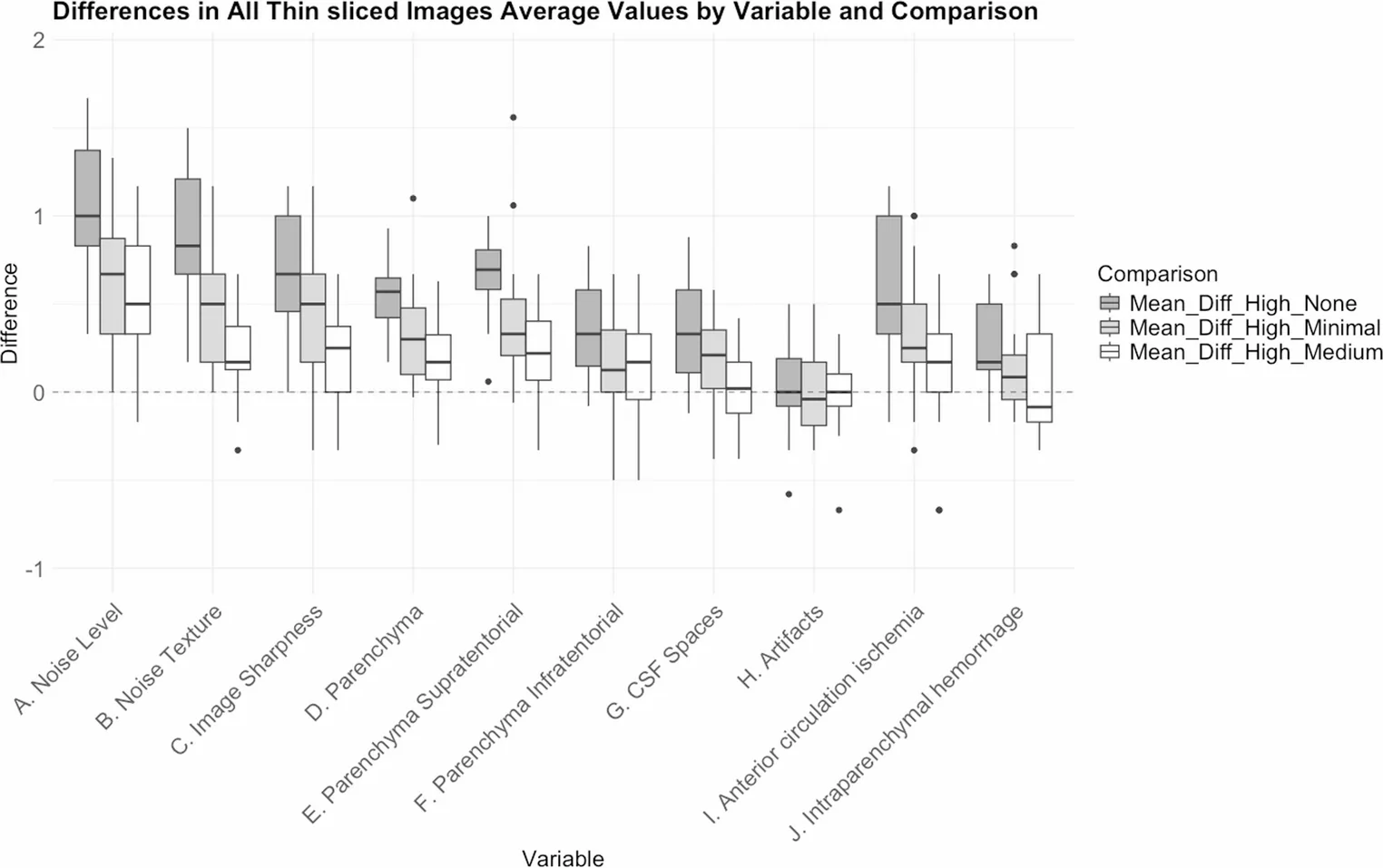
Boxplots showing distributions of paired thin-slice Likert score differences between High-level denoising and each comparator across readers and examinations. Positive values favor High-level denoising. Comparators are the standard reconstruction without added deep-learning denoising (dark gray), Minimal (light gray), and Medium (white). Center lines indicate medians, boxes interquartile ranges, and whiskers extend to 1.5 times the interquartile range. Exact adjusted *p* values are reported in Supplemental Table 3

Relative to the standard reconstruction without additional denoising, median paired thin-slice score improvements with High-level denoising were 1.00 for perceived noise level, 0.83 for noise texture, 0.67 for sharpness, 0.57 for overall brain parenchyma, 0.33 for cerebrospinal-fluid spaces, 0.50 for confidence in assessing anterior-circulation ischemia, and 0.17 for confidence in assessing intraparenchymal hemorrhage (all adjusted *p <* = 0.003). Perceived artifact severity did not differ (median difference 0.00, adjusted *p*>.99). Thin-slice distributions are shown in Fig. 5 and thick-slice distributions in Supplemental Fig. 1; exact adjusted* p *values for all three High-level contrasts are reported in Supplemental Table 3.

Ordinal-weighted Gwet AC2 coefficients ranged from 0.622 (95% CI, 0.546–0.664) for image sharpness to 0.802 (95% CI, 0.742–0.845) for infratentorial parenchyma in thin slices, and from 0.672 (95% CI, 0.641–0.699) for perceived artifact severity to 0.800 (95% CI, 0.744–0.845) for infratentorial parenchyma in thick slices (Supplemental Table 4 for details), representing generally substantial inter-rater agreement.

Site-specific distributions were directionally similar and are shown in Supplemental Figs. 2 and 3. Some median score differences were numerically larger at Karolinska University Hospital than at St Michael's Hospital. These exploratory observations were not tested with a formal interaction, as center was confounded by acquisition trajectory.

## Discussion

This retrospective multicenter cohort study evaluated whether a deep-learning-based denoising algorithm improved objective and subjective brain CBCT image quality relative to the standard reconstruction. High-level denoising improved all prespecified objective image-quality metrics and most subjective image-quality ratings. Similar directional findings were observed in the two site-specific datasets, but between-center differences were not formally tested.

Compared descriptively with published brain CT studies, gray- and white-matter noise in High-level denoised thick-slice CBCT was slightly lower than values reported with iterative or hybrid-iterative reconstruction [7, 19]. Gray-white matter CNR was numerically higher than in those reports and similar to that reported with DL reconstruction [12], while thin-slice CNR was within the range reported for thick-slice iterative or hybrid-iterative CT [7, 19]. These are indirect cross-study comparisons involving different populations, acquisitions, reconstructions, slice thicknesses, and ROI methods and therefore do not establish equivalence or superiority between CBCT and conventional CT. Nevertheless, the improved gray-white matter CNR is clinically relevant because tissue differentiation is important in acute stroke assessment.

Objective artifact indices decreased with increasing denoising strength, whereas no statistically significant difference in median perceived artifact-severity ratings was detected. Published conventional CT values provide descriptive cross-study context only [7, 12, 19]; because the present study did not include a direct conventional CT comparator, it cannot determine how artifact severity differs between CBCT and conventional CT.

The higher median ratings obtained with High-level denoising, generally 3–4, indicate improved perceived interpretability, whereas infratentorial ratings of 2–3 identify posterior-fossa assessment as an important remaining challenge. Although image-quality and reader-confidence ratings do not directly measure lesion-detection accuracy, they characterize clinically relevant dimensions of interpretability and represent an important step in evaluating a reconstruction method before diagnostic-accuracy testing. A prior study reported diagnostic performance of CBCT for ischemia and hemorrhage [7]. Although the present study did not directly test those accuracy estimates, it complements that evidence by demonstrating improvements across multiple objective and subjective image-quality measures and provides a strong rationale for adequately powered diagnostic-accuracy studies of DL denoising-enhanced CBCT in the interventional suite.

Image-quality measures generally improved with increasing denoising strength, consistent with prior reports of denoising-related improvements in CT [11–13]. Image-quality improvements appeared more pronounced for thin-slice than for thick-slice images, although denoising effects were not formally compared between slice thicknesses; the clinical implications, including for small or subtle findings, and the optimal reconstructed slice thickness remain uncertain [11, 13]. Alternative DL-based and classical denoising approaches have been reported [21, 22], including in a CBCT application [23]. A rigorous direct comparison would require the methods to be evaluated on common data using task-appropriate implementations and settings [21] and was beyond the scope of this study. Accordingly, the present findings are specific to the evaluated algorithm and settings and do not establish superiority over other denoising methods.

Key strengths of this study include the use of six experienced readers from two international centers, who performed blinded assessments of CBCT datasets acquired using both conventional circular and dual-axis trajectories. Limitations include the small sample (*n* = 20), lack of a formal sample-size calculation, and female predominance. The single randomized reading sequence did not force separation of alternative versions of the same examination, allowing possible recall effects. The study assessed one algorithm over standard reconstruction and did not include alternative commercial or other open-source denoising methods. Finally, image-quality ratings and diagnostic-confidence ratings do not establish diagnostic accuracy for ischemia or hemorrhage.

In conclusion, in a small retrospective multicenter cohort, High-level processing with a deep-learning-based denoising algorithm improved objective and subjective brain CBCT image-quality relative to the standard reconstruction without additional denoising. No difference in perceived artifact severity was detected. These findings support further evaluation of deep learning-enhanced cone-beam CT denoising for brain assessment in the interventional suite.

## Supplementary Information

Below is the link to the electronic supplementary material.

Supplementary Material 1

## Data Availability

Data generated or analyzed during the study are available from the corresponding author by request.

## Abbreviations

CBCT: Cone-Beam CT
CSF: Cerebrospinal Fluid
DL: Deep Learning
