# Supplementary material for: Brain cone-beam CT image quality improvement using a deep-learning-based denoising method: a multicenter retrospective study
Source: Neuroradiology. 2026 Aug 24;68(9):3083–91. doi: 10.1007/s00234-026-04148-9 (PMC13680336; doi:10.1007/s00234-026-04148-9)
Supplement: Supplementary file 1 [file 234_2026_4148_MOESM1_ESM.pdf]

Supplementary Information

Supplemental Table 1. Scan Details

	**Karolinska University Hospital**	**St Michael's Hospital**
Tube kilovoltage (kV)	120	120
Rotation time (s)	20.0	8.0
Trajectory	200° circular rotation	200° rotation +/- 15° amplitude CAUD/CRAN angulation
Nominal beam width (mm)	194.7	194.7
Display FOV coronal x sagittal x axial (mm^3)	251.8 x 251.8 x 194.7	251.8 x 251.8 x 194.7
Minimum slice thickness (mm)	0.66	0.66
Image matrix size	512 x 384	512 x 384
Radiation dose (CTDI, 16 cm phantom)	45 mGy	45 (n=8) or 65 (n=2) mGy

Supplemental Table 2. All Denoising Levels

Metric and slice thickness	High	Medium	Minimal	None
Gray matter noise				
Thick (5 mm)	2.33 (0.39)	3.04 (0.47)*	3.78 (0.58)*	4.54 (0.70)*
Thin (0.66 mm)	4.04 (0.61)	5.51 (0.83)*	6.99 (1.07)*	8.48 (1.34)*
White matter noise				
Thick (5 mm)	2.15 (0.41)	2.93 (0.53)*	3.73 (0.67)*	4.55 (0.81)*
Thin (0.66 mm)	3.78 (0.62)	5.36 (0.90)*	6.39 (1.19)*	8.52 (1.48)*
Gray matter SNR				
Thick (5 mm)	15.68 (4.73)	11.85 (3.47)*	9.53 (2.76)*	7.94 (2.29)*
Thin (0.66 mm)	9.10 (2.77)	6.63 (1.94)*	5.23 (1.50)*	4.32 (1.23)*
White matter SNR				
Thick (5 mm)	13.63 (4.84)	9.66 (3.33)*	7.39 (2.67)*	6.11 (2.07)*
Thin (0.66 mm)	7.58 (2.91)	5.24 (1.98)*	4.02 (1.51)*	3.25 (1.22)*
Gray vs white matter CNR				
Thick (5 mm)	3.86 (0.84)	2.89 (0.63)*	2.29 (0.51)*	1.91 (0.42)*
Thin (0.66 mm)	2.31 (0.39)	1.67 (0.29)*	1.31 (0.24)*	1.08 (0.20)*
Subcalvarial artifact index				
Thick (5 mm)	5.88 (2.03)	6.17 (2.04)*	6.53 (2.04)*	6.95 (2.05)*
Thin (0.66 mm)	6.73 (2.05)	7.52 (2.03)*	8.44 (2.05)*	9.46 (2.11)*
Posterior fossa artifact index				
Thick (5 mm)	4.77 (1.63)	5.54 (1.62)*	6.39 (1.65)*	7.31 (1.71)*
Thin (0.66 mm)	7.21 (2.03)	8.85 (2.09)*	10.59 (2.23)*	12.41 (2.43)*

Note: Data are mean (SD). None denotes the standard reconstruction without added deep-learning denoising. “Subcalvarial” denotes a region adjacent to the skull at the basal ganglia level. Noise and artifact indices are in Hounsfield units (HU); SNR and CNR are unitless. For each objective metric and slice format, High denoising was compared separately with the standard reconstruction (“None”), Minimal denoising, and Medium denoising using two-sided paired t tests. Bonferroni adjustment was

applied across these three comparisons within each metric and slice format. All two-sided Bonferroni-adjusted p values were <.001, denoted with an * in the table.

Supplemental Table 3. Pairwise p values for subjective ratings

**Outcome**	Slice	High vs None	High vs Minimal	High vs Medium
Noise Level	Thin	<0.001	<0.001	<0.001
	Thick	<0.001	0.001	0.006
Noise Texture	Thin	<0.001	<0.001	0.025
	Thick	0.001	0.037	0.260
Image Sharpness	Thin	<0.001	0.002	0.019
	Thick	0.058	1.000	1.000
Parenchyma	Thin	<0.001	<0.001	0.024
	Thick	0.004	1.000	0.244
Parenchyma Supratentorial	Thin	<0.001	<0.001	0.032
	Thick	<0.001	0.185	0.076
Parenchyma Infratentorial	Thin	<0.001	0.026	0.204
	Thick	0.477	0.667	1.000
CSF Spaces	Thin	<0.001	0.023	0.935
	Thick	0.031	1.000	0.518
Perceived artifact severity	Thin	1.000	1.000	1.000
	Thick	0.203	0.154	0.510
Anterior Circulation Ischemia	Thin	<0.001	0.005	0.309
	Thick	0.077	0.200	0.405
Intraparenchymal Hemorrhage	Thin	0.003	0.085	0.746
	Thick	0.146	1.000	1.000

Note: Two-sided Bonferroni-adjusted p values for the three prespecified High-level contrasts within each outcome and slice format. Ordinal ratings were compared using Wilcoxon matched-pairs signed-rank tests.

Supplemental Table 4. Interreader agreement for subjective ratings by slice format

**Outcome**	**Thin (0.66 mm), AC2 (95% CI)**	**Thick (5 mm), AC2 (95% CI)**
Noise level	0.693 (0.630–0.738)	0.712 (0.676–0.743)
Noise texture	0.628 (0.563–0.680)	0.672 (0.631–0.710)
Image sharpness	0.622 (0.546–0.664)	0.691 (0.656–0.725)
Overall brain parenchyma	0.721 (0.680–0.759)	0.753 (0.725–0.781)
Supratentorial parenchyma	0.702 (0.647–0.741)	0.767 (0.719–0.798)
Infratentorial parenchyma	0.802 (0.742–0.845)	0.800 (0.744–0.845)
CSF spaces	0.647 (0.610–0.687)	0.687 (0.640–0.734)
Perceived artifact severity	0.642 (0.599–0.674)	0.672 (0.641–0.699)
Confidence in assessing anterior-circulation ischemia	0.668 (0.605–0.711)	0.750 (0.711–0.784)
Confidence in assessing intraparenchymal hemorrhage	0.634 (0.581–0.681)	0.717 (0.648–0.764)

Note: Values are ordinal-weighted multi-rater Gwet AC2 coefficients (95% confidence intervals) for the six readers. Ratings from all four reconstruction conditions were included. For outcomes assessed at multiple anatomic locations, the original location-level 1–5 ratings were pooled. Ordinal weights were 1.00, 0.90, 0.70, 0.40, and 0 for disagreements of 0–4 categories, respectively. Confidence intervals were obtained from 20,000 bias-corrected and accelerated bootstrap resamples clustered by examination.

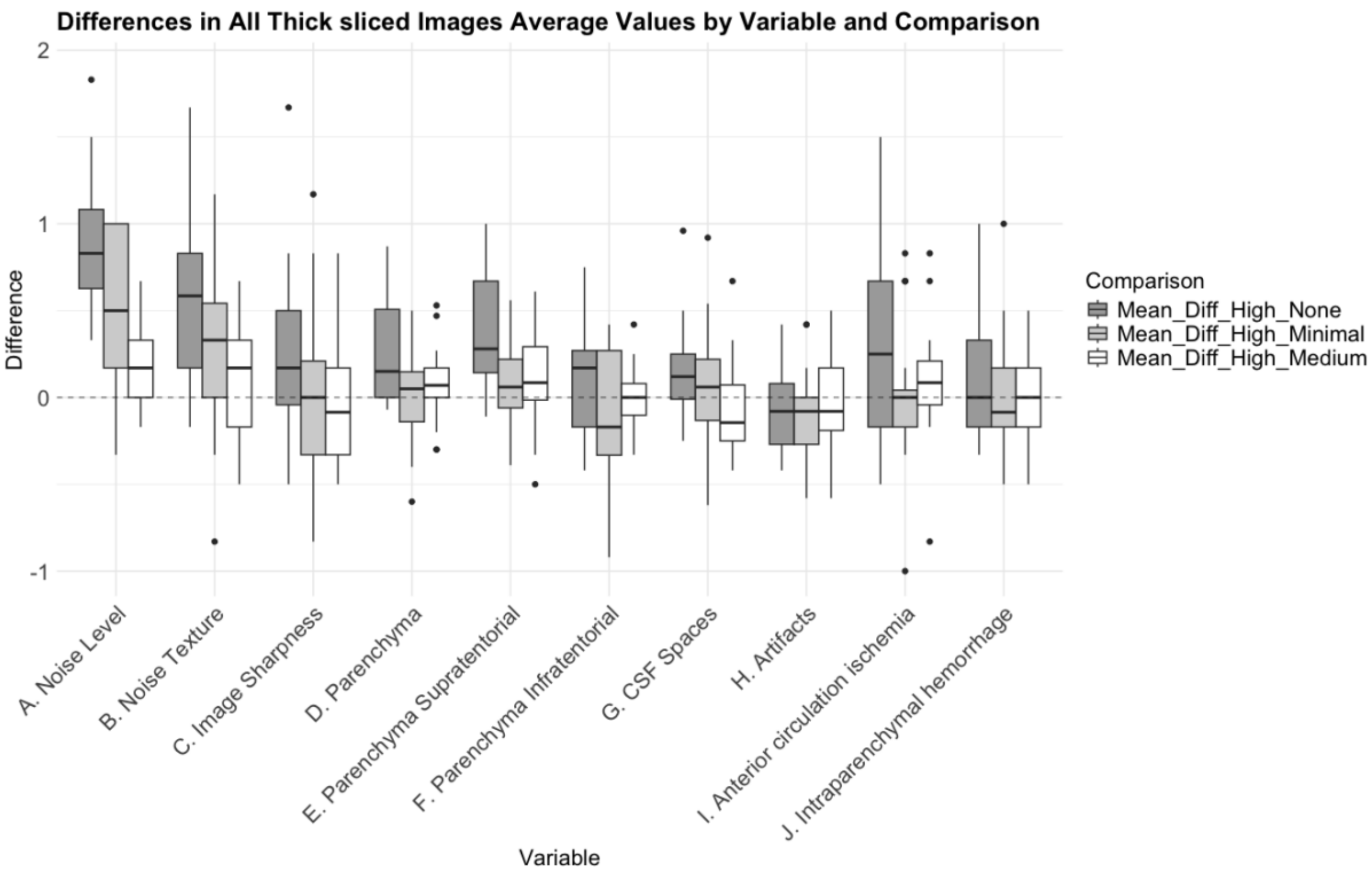

Supplemental Figure 1. Boxplots showing distributions of paired thick-slice Likert score differences between High-level denoising and the standard reconstruction without added deep-learning denoising (dark gray), Minimal denoising (light gray), and Medium denoising (white) across readers and examinations. Positive values favor High-level denoising. Center lines indicate medians, boxes interquartile ranges, and whiskers extend to 1.5 times the interquartile range.

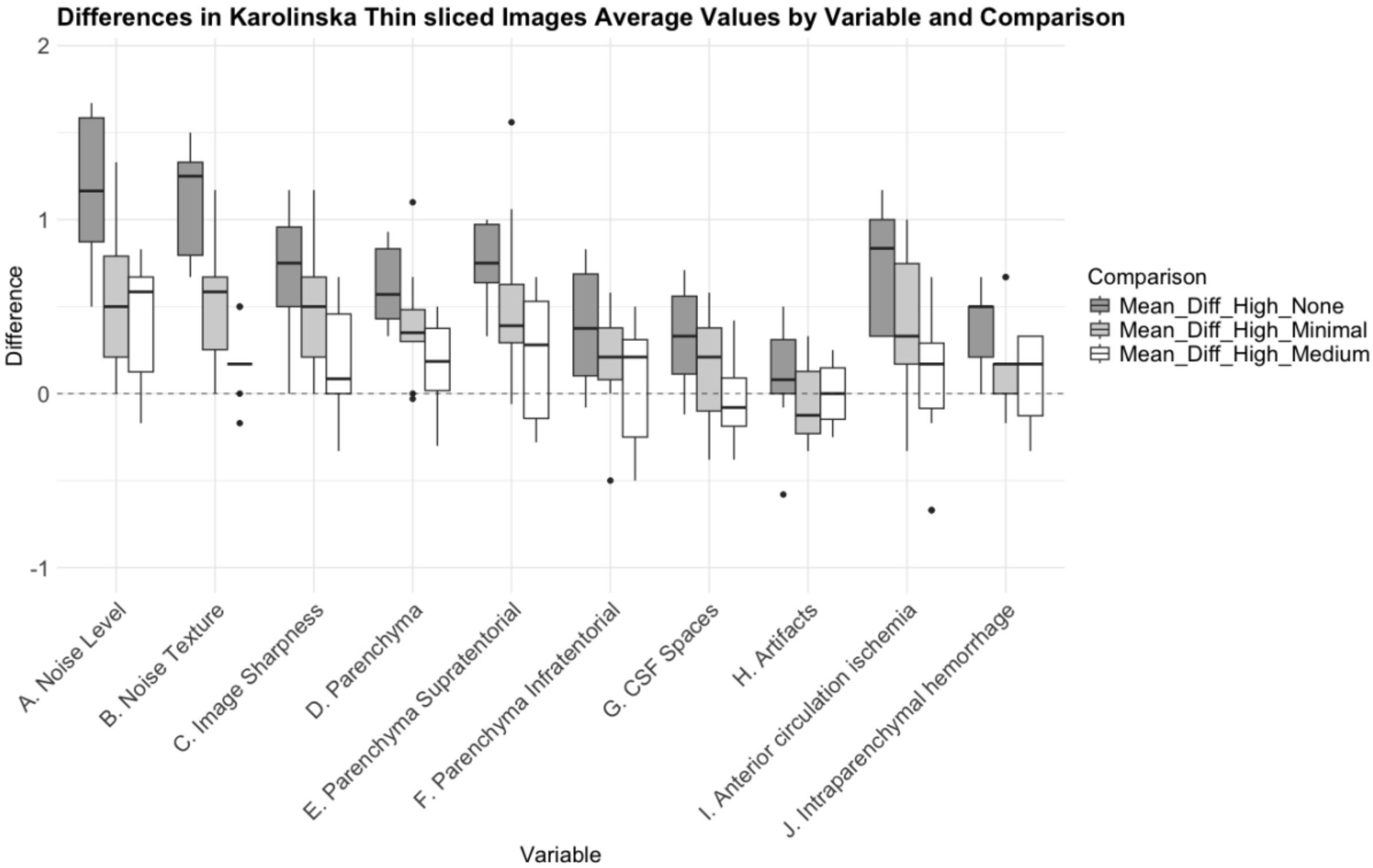

Supplemental Figure 2. Karolinska University Hospital thin-slice distributions of paired Likert score differences between High-level denoising and each comparator across readers and examinations. These site-specific results are descriptive; no formal between-center interaction was tested.

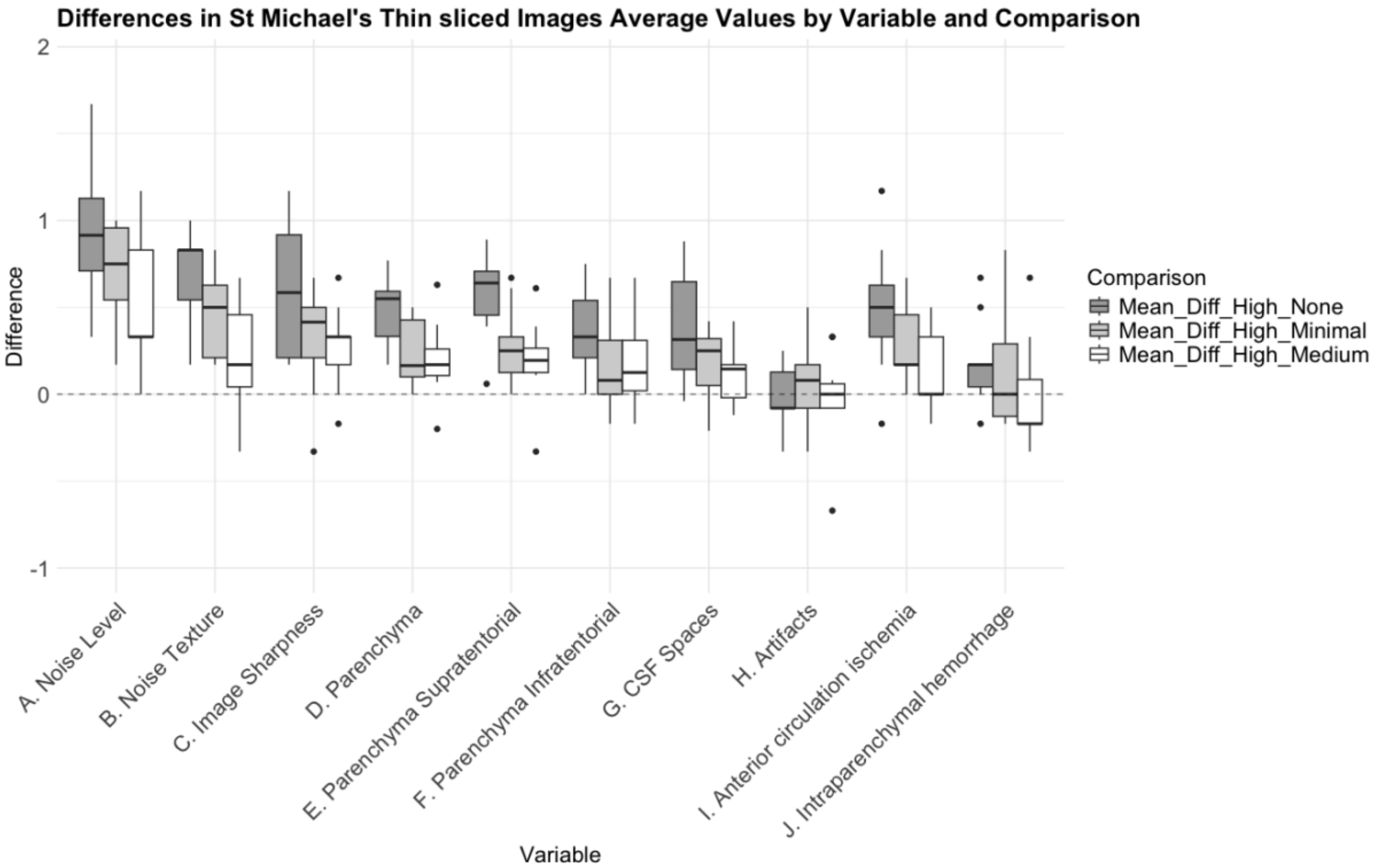

Supplemental Figure 3. St Michael’s Hospital thin-slice distributions of paired Likert score differences between High-level denoising and each comparator across readers and examinations. These site-specific results are descriptive; no formal between-center interaction was tested.

DL-based denoising algorithm

The herein used DL algorithm was exclusively trained on clinical CBCT data, including an even mixture of neuro scans with circular and helical acquisition trajectories. These were also acquired with 120kV tube voltage, as the clinical test data from the two sites in this study. Supervised training data pairs were then generated by simulating noise on top of normal dose as-is clinical acquisitions to obtain low-dose reconstructions. This results in matching pairs of low-dose and normal-dose reconstructions. These data pairs form the training data pairs for the supervised deep learning training. Full architecture and training details were proprietary and unavailable to the investigators, limiting independent reproducibility and direct comparison with other denoising implementations.

The DL-based denoising algorithm is trained to predict the noise in a given CBCT image volume x. We denote this noise prediction as a residual noise volume r. In this way, the application of this algorithm can be abstractly denoted as the functional mapping from an (noisy) input volume to its predicted residual noise volume r as

$$f(x) = r.$$

In this setting we define the herein differently parametrized denoising strengths (Minimal, Medium, and High) by using different linear scaling values before subtracting the estimated residual from the noisy input. We obtain a denoised volume $\hat{x}$ by using

$$\hat{x} = x - \lambda \cdot r.$$

In the context of this work the three used denoising strengths correspond to different scaling factors, as specified in the table below.

Denoising Strength	Scaling factor λ
Minimal	1/3
Medium	2/3
High	1

Objective image quality assessment details

ROIs were matched to an identical location in each scan. Noise in gray and white matter was calculated as the mean standard deviation of the corresponding ROIs. The same calculation was applied for the ROIs adjacent to the skull bone and posterior fossa, but due to the impact of beam hardening artifacts by the adjacent skull bone, these numbers are referred to as artifact indices. SNR was calculated as the mean CT number divided by the mean standard deviation (noise) of the ROIs. CNR between gray and white matter was calculated according to,

$$\text{CNR}(GM, WM) = \frac{\overline{GM} - \overline{WM}}{\sqrt{((\text{SD}(GM)^2 + \text{SD}(WM)^2)/2)}}$$

where $\overline{GM}$ and $\overline{WM}$ indicate the mean CT numbers and SD(*GM*) and SD(*WM*) are the standard deviations.

Supplemental Likert Questionnaire

"What is your impression of…?"

a. **The depiction of brain parenchyma including gray and white matter separation**
    i. Supraganglionic supratentorial cortex
    ii. Ganglionic supratentorial cortex
    iii. Basal ganglia
    iv. Brainstem
    v. Cerebellum
        1. Poor, uncertain for diagnosis
        2. Slight, limited diagnostic
        3. Decent, diagnostic
        4. Good, diagnostic
        5. Excellent, fully diagnostic

b. **The delineation of CSF spaces**
    i. Subcalvarial subarachnoid space
    ii. Supratentorial ventricles
    iii. 4th ventricle
    iv. Basal cisterns
        1. Poor, uncertain for diagnosis
        2. Slight, limited diagnostic
        3. Decent, diagnostic
        4. Good, diagnostic
        5. Excellent, fully diagnostic

c. **Artifacts**
    i. Supratentorial
    ii. Infratentorial
        1. Extensive artifacts, diagnostic evaluation almost impossible
        2. Moderate artifacts, diagnostic evaluation impaired
        3. Slight artifacts, may impair diagnostic evaluation
        4. Faint artifacts, likely does not impair diagnostic evaluation
        5. No artifacts

d. **General perception**
    i. Noise level
        1. High noise level
        2. .
        3. Moderate noise level
        4. .
        5. Low noise level

    ii. Noise texture
        1. Undesired texture, affecting diagnostic confidence
        2. .
        3. Acceptable texture, not affecting diagnostic confidence
        4. .
        5. Optimal/excellent texture, facilitating image assessment

iii. Image sharpness / perceived spatial resolution

1. Substantial undesired blurring of structures, non-diagnostic
2. .
3. Slight undesired blurring of anatomical structures, but not affecting diagnostic confidence
4. .
5. Excellent perception of anatomical structures, distinct separation of structures

iv. General impression of image quality to diagnose stroke

1. Poor, uncertain for diagnosis
2. Limited diagnostic quality
3. Decent, likely diagnostic
4. Good, diagnostic
5. Excellent, fully diagnostic
